# Psychological Distress, Skipped Meals, and Insufficient Sleep, and the Occurrence of Back Pain in Adolescent Female Soccer Players: The Karolinska Football Injury Cohort Study

**DOI:** 10.1177/19417381251326498

**Published:** 2025-03-27

**Authors:** Luca Orzali, Martin Asker, Nathan Weiss, Clara Onell, Urban Johnson, Anne Fältström, Ulrika Tranaeus, Eva Skillgate

**Affiliations:** Musculoskeletal and Sports Injury Epidemiology Center, Department of Health Promotion Science, Sophiahemmet University, Stockholm, Sweden; Musculoskeletal and Sports Injury Epidemiology Center, Department of Health Promotion Science, Sophiahemmet University, Stockholm, Sweden, and Naprapathögskolan - Scandinavian College of Naprapathic Manual Medicine, Stockholm, Sweden; Musculoskeletal and Sports Injury Epidemiology Center, Department of Health Promotion Science, Sophiahemmet University, Stockholm, Sweden, and Unit of Intervention and Implementation Research on Worker Health, Institute of Environmental Medicine, Karolinska Institutet, Stockholm, Sweden; Musculoskeletal and Sports Injury Epidemiology Center, Department of Health Promotion Science, Sophiahemmet University, Stockholm, Sweden; Halmstad University, Halmstad, Sweden; Unit of Physiotherapy, Department of Health, Medicine and Caring Sciences, Linköping University, Linköping, Sweden, and Region Jönköping County, Rehabilitation Centre, Ryhov County Hospital, Jönköping, Sweden; Unit of Intervention and Implementation Research on Worker Health, Institute of Environmental Medicine, Karolinska Institutet, Stockholm, Sweden, and Department of Physiology, Nutrition, Biomechanic, Sport Performance & Exercise Research and Innovation Center - Stockholm, SPERIC-S, The Swedish School of Sport and Health Sciences, Stockholm, Sweden; Musculoskeletal and Sports Injury Epidemiology Center, Department of Health Promotion Science, Sophiahemmet University, Stockholm, Sweden, Naprapathögskolan - Scandinavian College of Naprapathic Manual Medicine, Stockholm, Sweden, and Unit of Intervention and Implementation Research on Worker Health, Institute of Environmental Medicine, Karolinska Institutet, Stockholm, Sweden

**Keywords:** adolescent, back pain, cohort study, football, insufficient sleep, psychological distress

## Abstract

**Background:**

There is growing awareness that back pain in adolescent soccer (European football) players is not always related to local tissue damage. Approaches taking into consideration lifestyle factors are needed. This study aimed to investigate the association between psychological distress, skipped meals, and insufficient sleep, and the occurrence of back pain among adolescent female soccer players.

**Hypothesis:**

Psychological distress, skipped meals, and insufficient sleep are associated with the occurrence of back pain.

**Study Design:**

Cohort study.

**Level of Evidence:**

Level 3.

**Methods:**

Swedish adolescent female soccer players aged 12 to 17 years, without back pain at baseline, were included. The exposures psychological distress, skipped meals, and insufficient sleep were assessed at baseline. The players were followed for 1 year with a weekly web-based survey, where back pain intensity was measured on a numeric rating scale (NRS) ranging from 0 to 10. The outcome back pain was defined as reporting low back pain and/or upper back/neck pain intensity of ≥4 on the NRS. Multivariable Cox proportional hazard regression analyses were performed to estimate the hazard rate ratio (HRR) with 95% CI for the association between each exposure and the outcome back pain, adjusted for age at baseline and parents’ education.

**Results:**

In total, 351 players were included, and 141 players reported back pain at some point during the 1-year follow-up. The adjusted HRR for back pain was 1.79 (95% CI, 1.11-2.91) for insufficient sleep, 1.45 (95% CI, 0.97-2.17) for psychological distress, and 0.98 (95% CI, 0.61-1.59) for skipped meals.

**Conclusion:**

Insufficient sleep and psychological distress are associated with the occurrence of back pain among female adolescent soccer players.

**Clinical Relevance:**

Our results contribute to the understanding of what may influence the occurrence of back pain in adolescence, offering potential strategies for the prevention of back pain in adolescent female soccer players.

Back pain is common among adolescents, with a point prevalence ranging from 3% to 40% and a 1-month prevalence from 10% to 36%.^19,22^ Participation in competitive sports during adolescence may predispose to low back pain, with the prevalence varying considerably.^53^ Regarding soccer (European football), the lifetime prevalence of low back pain among adolescent players is between 34% and 50%,^11,15,21,44^ and female players have a higher risk of developing low back pain than male players.^17^ Neck pain has also been shown to be a common complaint among adolescent players, with a lifetime prevalence of 30%.^15^ Back pain significantly impacts the player’s career, causing prolonged absence from the field and early retirement.^12,46^ Understanding what determines back pain during adolescence is therefore crucial for taking preventive measures and avoiding future negative consequences for the player.

The cause of back pain in athletes is unclear and potential causes include, among others, direct trauma, muscle impairment, training overload, lumbosacral sprain, and muscular contusion.^51,56^ However, recent studies suggest that many adolescent soccer players suffer from back pain without any recognized underlying external cause or condition.^36,41^ There is a growing awareness that back pain in adolescence is a multidimensional condition and not always related to local tissue pathology or damage.^31,32,42^

Besides specific pathologies, lifestyle and psychosocial factors may play a role in the development of back pain, which highlights the multifaceted nature of back pain in adolescence. Among the lifestyle factors, sleep deficits and skipped meals have been associated with a higher prevalence of back pain in adolescents of the general population.^4,5,55^ Moreover, an association between psychological distress and back pain has been found in the Swedish general population, with a notably higher prevalence of comorbidity in women and girls.^34^ This association has also been described in cross-sectional studies in adolescents.^2,8^

A multidimensional approach to back pain in adolescent female soccer players is suggested, taking into consideration other drivers of pain, such as mental health, sleep, and diet.^2,4,5,8,31,32,34,36,41,42,55^ To our knowledge, no longitudinal study has investigated lifestyle as potential risk factors for back pain in adolescent female soccer players. Furthermore, according to systematic reviews, there is a lack of high-quality studies investigating the occurrence, prevalence, and potential risk factors for back pain in adolescent athletes.^33,53^ Therefore, high-quality prospective studies with a large sample are warranted to study our hypotheses. We hypothesize that factors such as psychological distress and lifestyle are associated with the occurrence of back pain.

The aim of this study was to investigate whether psychological distress, skipped meals, and insufficient sleep are associated with the occurrence of back pain in adolescent female soccer players.

## Methods

This study is based on data from the Karolinska Football Injury Cohort study (KIC), described briefly below. For extensive information about methods as the study setting and eligibility criteria for the cohort participants, we refer to the published study protocol.^50^ KIC is a longitudinal study of female soccer players in the Swedish Divisions I and II, 12 to 17 years old from 27 teams. All participating players and their legal guardians were informed about the study and provided written informed consent. If players were <15 years old, it was mandatory for their legal guardians to provide written informed consent on their behalf. The study was conducted in accordance with the declaration of Helsinki and approved by the Swedish Ethical Review Authority (Dnr 2016/1251-31/4).

### Baseline Measurements

At baseline, players were asked to fill out an extensive questionnaire covering several topics including demographics, general health, lifestyle, stress, lower back and upper back/neck pain, soccer-related factors, and socioeconomic factors. The inclusion of study participants was performed consecutively from year 2016 to 2019.

### Exposures

#### Psychological Distress

Psychological distress was surveyed in the baseline questionnaire using the 12-item General Health Questionnaire (GHQ-12), a valid tool used widely to assess psychological distress and common symptoms of mental disorders. This self-reporting questionnaire addresses various areas, including anxiety, depressed mood, and loss of confidence during the preceding weeks.^27,37^ It is suitable for multiple age groups and different sexes, and it has been validated for the Swedish general population.^7,45^ Each of the 12 questions contains 4 alternative answers ranging from “more/better than usual” to “much less/much worse than usual,” or “not at all” to “much more than usual,” depending on the question. The answers were quantified using a bimodal scoring (0-0-1-1 with 0 indicating no distress for each question), giving a total added score of 12. A cut-off of ≥3 was used to denote moderate or severe psychological distress.^1,24,39,48^

#### Skipped Meals

Participants answered how often they usually skipped breakfast, lunch, and dinner, respectively. The answers alternatives were “never,” “rarely, few times per year,” “sometimes per month,” “several times per week,” and “every day.” The exposure skipped meals was defined as skipping ≥1 meal several times per week or every day.^14^

#### Insufficient Sleep

Participants were asked to answer the question “how many hours per night do you normally sleep?” Based on the recommended sleep duration for teenagers stated by the National Sleep Foundation, a cut-off score of <8 hours was used to identify an insufficient sleep duration for overall health and wellbeing.^18^

### Follow-up Measurements and Outcome

Participants were followed prospectively with a weekly web-based survey for 1 year. Among other things, they were asked to report the intensity of low back pain and upper back/neck pain in the past 7 days.^50^ Pain intensity was measured with an 11-point numeric rating scale (NRS) ranging from 0 (no pain) to 10 (worst imaginable pain) for low back pain and upper back/neck pain, respectively. The dichotomous outcome “back pain” was defined as experiencing low back pain intensity and/or upper back/neck pain intensity of ≥4 on the NRS.^6^

### Confounding Factors

Potential confounding factors for the association between the exposures and outcome were chosen a priori from the baseline questionnaire regarding previously published literature and theoretical plausibility and with the use of directed acyclic graphs. This was done to ascertain whether the factors could be mediators, moderators, colliders, or lie in the causal pathway.^49^

Factors considered as potential confounders were age at baseline (continuous) and parents’ education dichotomized into having ≥1 parent with a university degree/higher education or not.^30^

### Statistical Analyses

**Figure 1.**
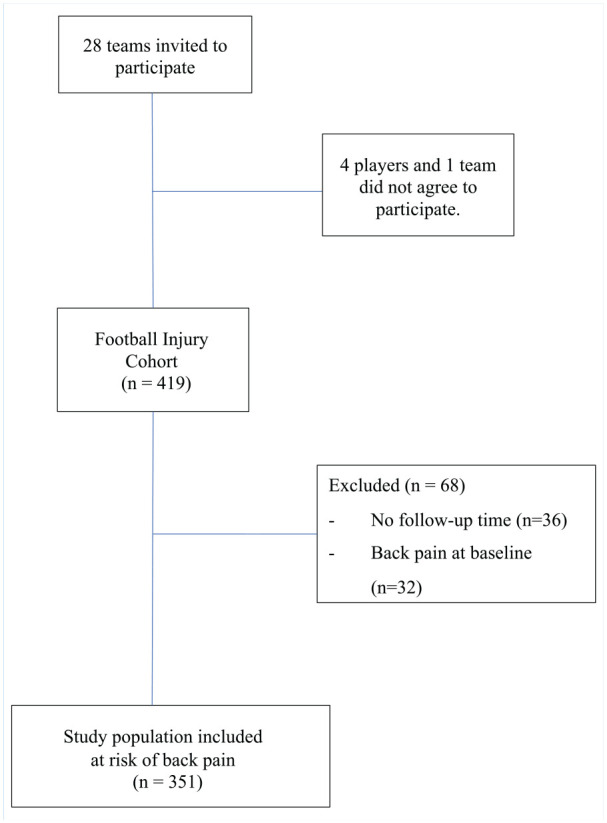
Flow diagram describing the inclusion of participants.

To study a population at risk, only participants without back pain (NRS ≤ 4) at baseline were included. Participants who had not answered any follow-up survey were excluded. The inclusion of participants is illustrated in Figure 1.

Descriptive analyses were performed to report the cohort characteristics at baseline and presented as means and standard deviations for continuous variables and as proportions for the categorical variables.

Back pain occurrence was calculated as the total number of participants who reported an event of back pain divided by the total follow-up times calculated as a sum of hours of training and matches.^38^ Kaplan-Meier (KM) curves were used to visualize the cumulative occurrence of back pain among exposed and unexposed.

To establish the association between exposures and the occurrence of back pain, a multivariable Cox proportional hazard model (with all exposures in the same model adjusted for each other) was used to calculate hazard rate ratio (HRR) and 95% CI. To assess a potential independent association between exposures and the occurrence of back pain, the multivariable model was also adjusted for the potential confounding factors described above (age at baseline and parents’ education).

The proportional hazards assumption was checked by visually inspecting the KM curves and the log (–log) versus log (time) plot.

Participants’ time at risk corresponded to the number of hours of soccer training and match play from the baseline until the first event of back pain, the end of the study (after 1 year) or until the player was censored. This information was collected in the weekly follow-up questionnaires. Censoring was performed if the player dropped out (decided to quit the study or stopped answering until the end of the study). Players who reported other injuries or other reasons for not participating in soccer (school break, illness, etc), or stopped answering for a while and then continued until the end of the study, were not censored.

Statistical analyses were performed with SPSS Version 28.0 (IBM Corp).

## Results

Table 1 summarizes the baseline characteristics of the 351 included players stratified by the exposures psychological distress, skipped meals, and insufficient sleep. The mean age of the total population was 13.3 (SD 1.1) years, ranging between 12 and17 years. A total of 76 players (22%) were exposed to psychological distress, 58 players (16%) to skipped meals, and 50 players (14%) to insufficient sleep.

**Table 1.** Baseline characteristics stratified by exposure

		Psychological Distress^*a*,*b*^		Skipped Meals^*c*^		Insufficient Sleep^*d*,*e*^	
Variables	Total Population n = 351 (100%)	Exposed n = 76 (22%)	Nonexposed n = 273 (78%)	Exposed n = 58 (16%)	Nonexposed n = 293 (84%)	Exposed n = 50 (14%)	Nonexposed n = 292 (83%)
Age, mean years (SD)	13.3 (1.1)	13.9 (1.1)	13.2 (1.0)	13.4 (1.2)	13.3 (1.1)	14.1 (1.1)	13.2 (1.0)
Height, mean cm (SD)^*f*^	162.7 (6.7)	163.7 (6.0)	162.4 (7.1)	163.0 (6.2)	162.6 (7.1)	164.9 (6.0)	162.3 (7.0)
Weight, mean kg (SD)^*f*^	53.0 (9.1)	55.1 (8.4)	52.4 (9.3)	55.6 (10.0)	52.4 (8.9)	58.0 (9.6)	52.0 (8.8)
Body mass index, mean kg/m^2^ (SD)^*b*^	19.9 (2.4)	20.4 (2.4)	19.7 (2.5)	20.9 (2.7)	19.7 (2.4)	21.2 (2.7)	19.6 (2.4)
Menarche, n (%)^*f*^	231 (66)	63 (83)	167 (62)	39 (67)	192 (66)	47 (94)	176 (61)
Years playing organized soccer, mean (SD)	7.0 (2.0)	7.5 (2.0)	7.0 (1.9)	7.0 (2.1)	7.1 (2.0)	8.1 (2.0)	6.8 (2.0)
Playing position, n (%)^*g*^							
Midfielder	148 (43)	33 (43)	115 (42)	24 (42)	124 (43)	14 (29)	131 (45)
Defender	110 (31)	27 (25)	82 (30)	17 (29)	93 (32)	17 (35)	90 (31)
Forward	65 (19)	10 (13)	54 (20)	12 (21)	53 (18)	12 (24)	52 (18)
Goalkeeper	25 (7)	6 (8)	19 (7)	5 (8)	20 (7)	6 (12)	18 (6)
Soccer matches, n/week, mean (SD)	1.5 (0.6)	1.3 (0.5)	1.5 (0.6)	1.4 (0.4)	1.5 (0.6)	1.5 (0.8)	1.5 (0.6)
Soccer training, hours/week, mean (SD)	4.9 (1.7)	4.9 (2.0)	4.9 (1.6)	5.0 (1.9)	4.9 (1.7)	5.0 (2.2)	4.8 (1.6)
Other soccer related training, hours/week, mean (SD)^*f*^	1.6 (1.4)	1.7 (1.2)	1.5 (1.4)	1.5 (1.2)	1.6 (1.4)	1.7 (1.9)	1.5 (1.2)
Educated parents, n (%)^*h*,*i*^	251 (72)	58 (81)	191 (75)	37 (68)	214 (78)	33 (67)	212 (77)

a Scoring ≥3 on the General Health Questionnaire 12 (GHQ-12).

b Missing values = 2 (1%).

c Skipping breakfast, lunch, and/or dinner several days per week or every day.

d Normally sleeping <8 hours per night.

e Missing values = 9 (3%).

f Missing values = 1 (<1%).

g Missing values = 3 (1%).

h Educated parents defined as having ≥1 parent with a university degree/higher education or not.

i Missing values = 22 (6%).

### Occurrence of Back Pain and Risk Analysis

During a total follow-up time of 40.367 hours of playing soccer, 141 players reported ≥1 event of back pain. This corresponded to an occurrence of 3.5 events of back pain per 1000 hours of playing soccer for all the included players.

**Figure 2.**
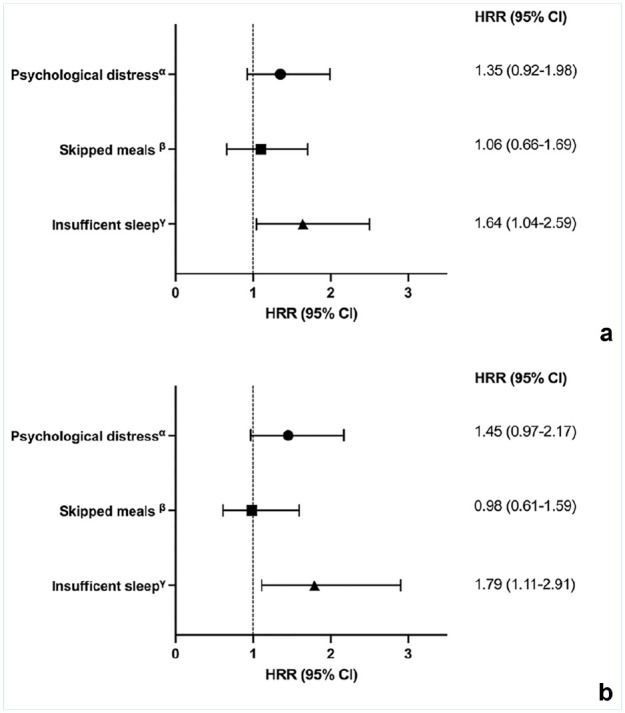
(a) Crude HRRs and 95% CI for the associations between psychological distress, skipped meals, insufficient sleep, and the occurrence of back pain. (b) HRRs and 95% CI adjusted for age and parents’ education. ^α^Scoring ≥3 on the GHQ-12. ^β^Skipping breakfast, lunch, and/or dinner several days per week or every day. ^γ^Normally sleeping <8 hours per night. GHQ-12, General Health Questionnaire 12; HRR, hazard rate ratio.

The adjusted HRR of back pain was for insufficient sleep 1.79 (95% CI, 1.11-2.91), for psychological distress 1.45 (95% CI, 0.97-2.17), and for skipped meals 0.98 (95% CI, 0.61-1.59), as presented in Figure 2.

**Figure 3.**
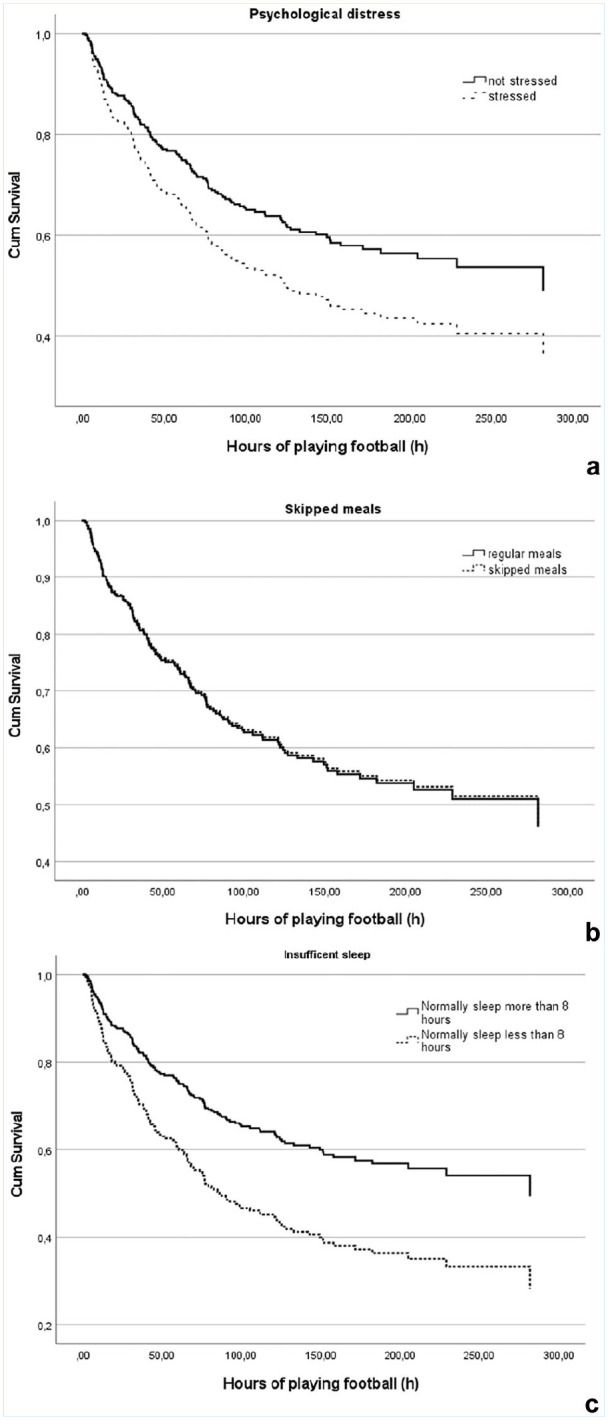
Adjusted Kaplan-Meier curves displaying exposed (a) psychological distress, (b) skipped meals, and (c) insufficient sleep versus nonexposed survival time related to back pain.

Adjusted KM curves from the multivariable Cox proportional hazard model are presented in Figure 3.

## Discussion

Our results showed that insufficient sleep, defined as normally sleeping <8 hours per night, was associated with a higher occurrence of back pain in female adolescent soccer players. Furthermore, being psychologically distressed was associated with a higher occurrence of back pain even if the adjusted confidence interval included 1. On the contrary, we did not find any association between skipped meals and the occurrence of back pain.

In agreement with our findings, other studies found poor sleep duration (<8.5 hours of sleeping) to be associated with back pain in the general population of adolescents.^10,54^

There might be several possible mechanisms for the association between insufficient sleep and back pain in adolescent female soccer players. Among adolescent soccer players, later bedtimes are reasonably common when training and matches are performed in the evening. Consequently, the need to get up early to go to school may result in fewer hours spent in bed than recommended. Moreover, natural variations of training load may disturb the biological circadian rhythm, which can impact sleep duration.^10,26^ Insufficient sleep can impair the inhibitory function, decreasing the pain tolerance and the pain thresholds. This could make the athlete vulnerable to joint overload, enhancing painful stimuli and fatigue, leading to back pain.^3,25^ A study on female adolescent soccer players emphasized the importance of adequate sleep duration in mitigating the negative effects of training load on their wellbeing. Decreased sleep duration intensified the adverse impact of training load on their wellbeing, while an increased sleep duration minimized the negative impact.^54^ In turn, lower ratings of subjective wellbeing (such as mood or fatigue) are known to be associated with increased pain.^13,20^

Our results indicate a higher occurrence of back pain for players who are psychologically distressed. To the best of our knowledge, among young soccer players, there are no other studies investigating the relationship between psychological distress and the occurrence of back pain. Similarly, a study showed a strong association between mental health problems, such as stress and depression, and back pain among the general population of 14 year olds.^40^

Among young athletes, many studies have found an association between insufficient sleep or psychological distress, and an increased risk of any kind of injury.^29,35,47,52^ However, the term “injury” is often explained as pain with reduced training volume, difficulties participating in normal training or competition, and reduced performance.^9^ Our outcome is not injury but moderate or severe back pain for any reason, making it difficult to compare our findings with studies of sports injuries in the back.

Skipped meals is common among adolescents, and breakfast appeared to be the meal skipped the most.^16,23^ In our cohort, 16% skipped meals, and breakfast was also the most skipped meal (12%). However, we did not find any associations between skipped meals and back pain in our study. Contrary to our findings, a recent cross-sectional study identified an association between skipping breakfast and back pain in a general population of children and adolescents.^5^ However, the cross-sectional study design hampers conclusions about a causal relationship.

### Methodological Discussion and Limitations

Our study does not assume causality but focuses on the association between the exposures and the outcome. However, excluding participants with back pain at baseline, combined with the prospective study design allows us to suggest a possible direction for this association. The longitudinal study design with a healthy cohort at risk of developing back pain, taking into consideration relevant confounding factors, is therefore a methodological strength. Still, there is a risk of unmeasured and residual confounding from differences between exposed and nonexposed participants.

To be able to assess the potential impact of changing exposure status over time, a questionnaire with similar content as the baseline questionnaire was sent out after 1 year with a response rate of 66% in this study population. After 1 year, 37% were exposed to psychological distress, and 21% skipped meals. The corresponding figures at baseline were 22% and 17%, indicating an increase over time. We do not know whether players lost to follow-up in this 1-year questionnaire differed from those who answered regarding the exposure status, but we judge the associations in our study not to be overestimated because of changes in exposure status over time.

Regarding the classification of the outcome, we chose a cut-off of ≥4 on the NRS to be classified as having back pain. We cannot rule out that the results of the study could be different if other cut-offs were chosen. However, in patients with chronic musculoskeletal pain, the cut-off 4 has been found to represent moderate pain,^6^ and in children with chronic pain NRS is suggested to be a valid measure for assessing pain intensity.^43^ Further, this cut-off gave us sufficient statistical power for the calculations of the associations between the exposures and the outcome.

Psychological distress was assessed with the instrument GHQ-12 that present good validity and reliability in the Swedish population.^27,28,45^ The fact that sleep hours, skipped meals, and psychological distress were self-reported might result in a misclassification of the exposure given that a perception of social desirability may drive an under or overestimation of lifestyle behaviors. However, since the study design is prospective, the risk of a differential misclassification, and, thereby, a biased result due to this potential limitation, is low.

At 1 year follow-up, the response rate of the weekly web-based survey was as high as 74%, which minimizes the risk of selection bias. We judge that the study has good external validity given the large sample size, which is considered representative of adolescent female soccer players in Sweden. The results are likely generalizable to other team-sport female adolescent athletes with similar training loads and sports demands.

## Conclusion

Insufficient sleep and psychological distress are associated with a higher occurrence of back pain among female adolescent soccer players.

## Clinical Relevance

Back pain is a common and troublesome clinical problem for female adolescent soccer players. Our results contribute to the understanding of which factors may influence the occurrence of back pain in adolescence, offering potential strategies for the prevention of back pain in adolescent female soccer players.
